# Volatiles from the hypoxylaceous fungi *Hypoxylon griseobrunneum* and *Hypoxylon macrocarpum*

**DOI:** 10.3762/bjoc.14.277

**Published:** 2018-12-04

**Authors:** Jan Rinkel, Alexander Babczyk, Tao Wang, Marc Stadler, Jeroen S Dickschat

**Affiliations:** 1 Kekulé-Institut für Organische Chemie, Universität Bonn, Gerhard-Domagk-Straße 1, 53121 Bonn, Germany; 2 Abteilung Mikrobielle Wirkstoffe, Helmholtz-Zentrum für Infektionsforschung, Inhoffenstraße 7, 38124 Braunschweig, Germany

**Keywords:** constitutional isomerism, gas chromatography, mass spectrometry, natural products, volatiles

## Abstract

The volatiles emitted by the ascomycetes *Hypoxylon griseobrunneum* and *Hypoxylon macrocarpum* (Hypoxylaceae, Xylariales) were collected by use of a closed-loop stripping apparatus (CLSA) and analysed by GC–MS. The main compound class of both species were polysubstituted benzene derivatives. Their structures could only be unambiguously determined by comparison to all isomers with different substitution patterns. The substitution pattern of the main compound from *H. griseobrunneum*, the new natural product 2,4,5-trimethylanisole, was explainable by a polyketide biosynthesis mechanism that was supported by a feeding experiment with (*methyl*-^2^H_3_)methionine.

## Introduction

**Figure 1:**
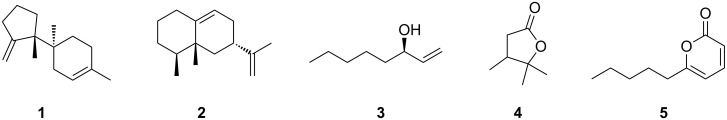
Structures of fungal volatiles. Trichodiene (**1**), aristolochene (**2**), (*R*)-oct-1-en-3-ol (**3**), 3,4-dimethylpentan-4-olide (**4**), and 6-pentyl-2*H*-pyran-2-one (**5**).

Fungi release a large number of different volatiles that belong to all kinds of natural product classes [1]. Many of these compounds are of interest, because they are markers for the production of fungal toxins and thus can help to distinguish between toxigenic and closely related non-toxigenic species. For example, the sesquiterpene trichodiene (**1**, Figure 1) is the precursor of the trichothecene family of mycotoxins [2], a class of highly bioactive secondary metabolites that belong to the strongest known inhibitors of protein biosynthesis in eukaryotes [3]. Similarly, the sesquiterpene aristolochene (**2**) is the parent hydrocarbon of PR toxin [4–5] and has been used as a marker to differentiate between toxin producing and non-producing *Penicillium roqueforti* isolates [6]. On the other hand, fungal volatiles are interesting, because they contribute with their aroma to the flavour of many edible mushrooms. One of the first identified and certainly most widespread compounds is matsutake alcohol, (*R*)-oct-1-en-3-ol (**3**), that is produced inter alia by *Tricholoma matsutake* [7], a highly sought delicacy in the Japanese cuisine, the bottom mushroom *Agaricus bisporus*, and the penny bun *Boletus edulis* [8], as the name indicates a European equivalent to Matsutake in high-class cooking. Volatile organic compounds are also important in the interaction between different species, e.g., between ophiostomatoid fungi and conifer bark beetles that show different behavioural responses to fungal volatiles [9]. Fungal volatiles can also be of importance in the interaction between plants and fungi. In some cases, fungal volatiles seem to be involved in the plant pathogenicity of fungi, as recently observed for 3,4-dimethylpentan-4-olide (**4**), a volatile from the ash pathogen *Hymenoscyphus fraxineus* that currently threatens the European ash population [10]. Both enantiomers of this lactone were found to inhibit ash seed germination and to cause necrotic lesions in the plant tissue. In other cases, fungal volatiles can have beneficial effects and may even be involved in the induction of systemic resistance in plants, as can be assumed for 6-pentyl-2*H*-pyran-2-one (**5**) that is produced by many fungi from the genus *Trichoderma* [11–12].

Fungal volatiles can be efficiently analysed by trapping, e.g., on charcoal filters with a closed-loop stripping apparatus (CLSA) that was developed by Grob and Zürcher [13], followed by filter extraction and GC–MS analysis of the obtained headspace extracts [14]. The unambiguous compound identification requires a good match of the recorded electron impact (EI) mass spectrum to a database spectrum and of the retention index, a standardised GC retention factor that is calculated from the retention times of the analytes and of *n*-alkanes [15], in comparison to an authentic standard or published data. A peculiar problem in the analysis of aromatic compounds with multiple substituents is that constitutional isomers with the same types of substituents, but different substitution patterns often have very similar mass spectra. Furthermore, some of the isomers may also have similar retention indices, and therefore it is mandatory for unambiguous structure elucidation to compare analytes that fall into this class to all the possible isomers. A similar problem can apply to the structural assignment of compounds with multiple stereocentres based on GC–MS data, because the various possible diastereomers usually also produce very similar mass spectra [16], a phenomenon that is also reported for *E* and *Z* stereoisomers and can lead to wrong structural assignments, if no authentic standards are used for comparison [17]. We have recently reported on two chlorinated aromatic compounds from an endophytic *Geniculosporium* sp. [18] and on a series of structurally related phenols, benzaldehydes and anisole derivatives from *Hypoxylon invadens* [19] that could only be identified with certainty following this approach of extensive compound comparisons. Members of the family Hypoxylaceae are regarded to be extremely rich in secondary metabolites [20], but not much is known about volatiles from these fungi [21]. In continuation of this work, here we present the volatiles emitted by *Hypoxylon griseobrunneum* MUCL 53754 and *Hypoxylon macrocarpum* STMA 130423. These strains were selected, because both species released a characteristic and strong odour, as was already mentioned in the literature for *H. macrocarpum* [22–23], but the nature of the odoriferous compounds remained unknown. As will be shown, the bouquets of both species are composed mainly of highly substituted aromatic compounds whose structures were only securely identifiable by comparison to all the possible constitutional isomers with different ring substitution patterns.

## Results and Discussion

### Headspace analysis

The volatiles released by agar plate cultures of *H. griseobrunneum* and *H. macrocarpum* were collected using a CLSA [13]. After a collection time of one day the charcoal filter traps were removed and extracted with CH_2_Cl_2_, followed by GC–MS analysis of the obtained extracts. For both strains a large number of compounds from different compound classes including alcohols, ketones, esters, terpenes and pyrazines were identified. Besides the observed minor production of compounds from these classes aromatic compounds dominated, but the patterns were strain-specific.

### Identification of volatiles from *Hypoxylon griseobrunneum*

**Figure 2:**
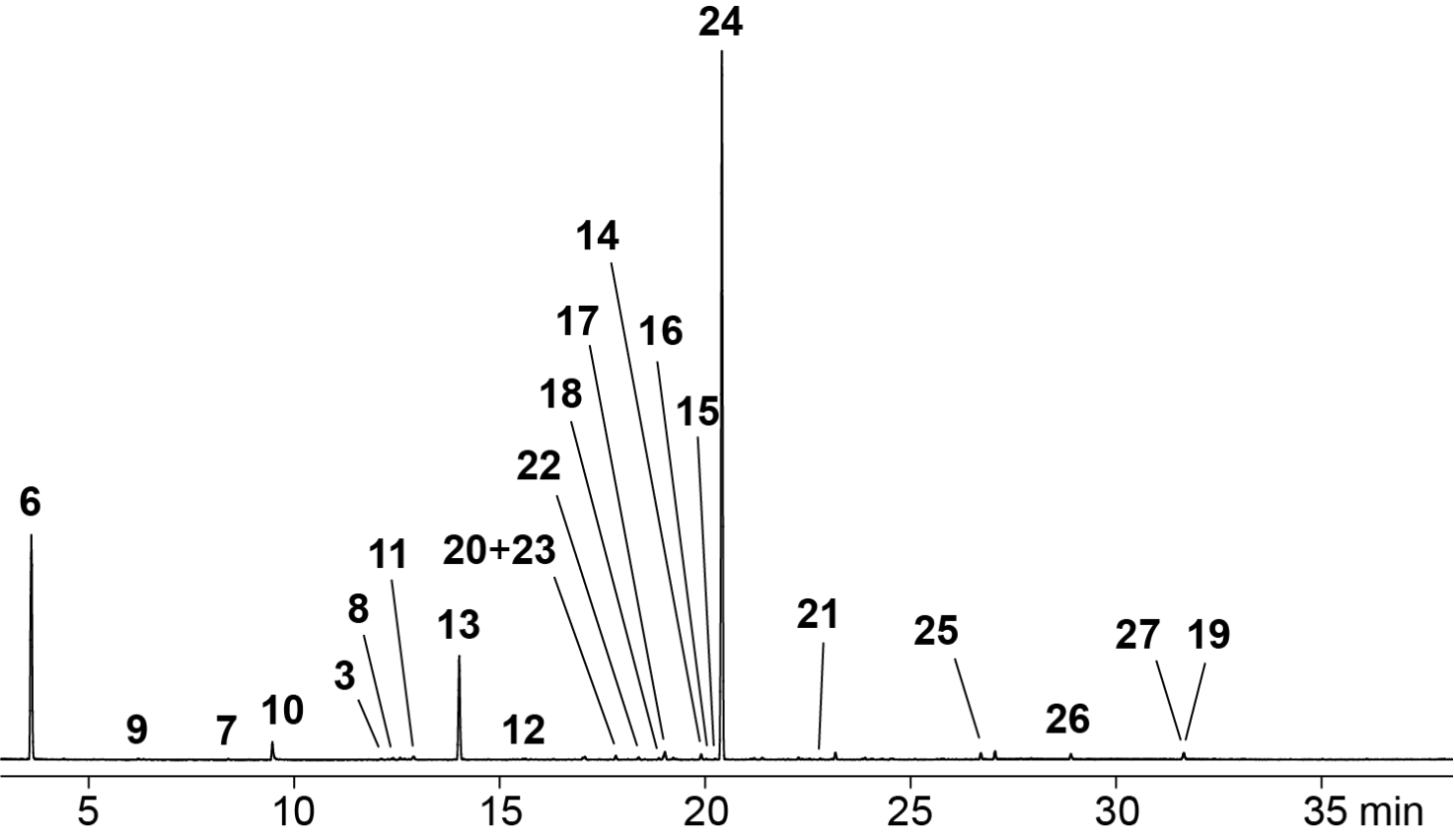
Total ion chromatogram of a CLSA headspace extract from *Hypoxylon griseobrunneum* MUCL 53754. Peak numbers refer to compound numbers in Table 1 and in Figure 3.

**Figure 3:**
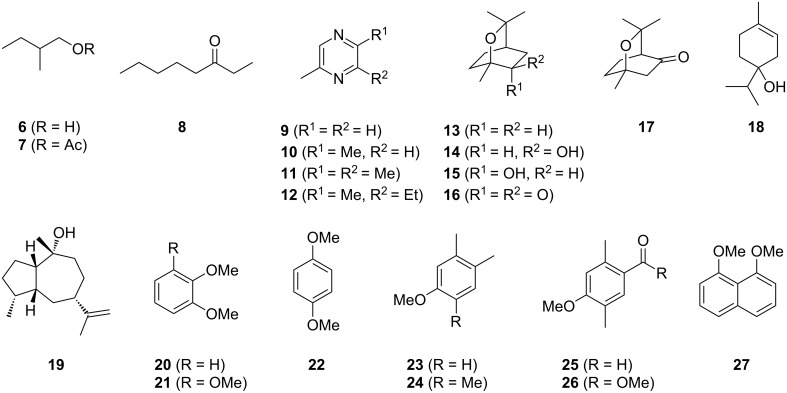
Volatiles from *Hypoxylon griseobrunneum*.

A representative total ion chromatogram for the volatiles released by *Hypoxylon griseobrunneum* is shown in Figure 2 and the results of the analysis are compiled in Table 1. Several compounds in the headspace extract were readily identified from their mass spectra and retention indices, including the widespread alcohol 2-methylbutan-1-ol (**6**) as one of the main compounds and traces of the corresponding acetate ester **7** (Table 1 and Figure 3). Small amounts of matsutake alcohol (**3**) were also found. This volatile is frequently accompanied by other C_8_ metabolites [1], which is reflected for *H. griseobrunneum* by the detection of octan-3-one (**8**). Trace amounts of a series of alkylated pyrazines including methylpyrazine (**9**), 2,5-dimethylpyrazine (**10**), trimethylpyrazine (**11**) and 2-ethyl-3,6-dimethylpyrazine (**12**) were also observed. These compounds were previously reported from the actinobacterium *Corynebacterium glutamicum* in which pyrazines are biosynthetically derived from acetoin and its higher homologs [24]. For unambiguous structure elucidation commercially available standards of **9**–**11** were used, while a synthesis of **12** was performed in our earlier study [24].

**Table 1:** Volatiles identified in the headspace extract from *Hypoxylon griseobrunneum* MUCL 53754.

compound	*I*^a^	*I* (lit.)	identification^b^	peak area^c^

2-methylbutan-1-ol (**6**)	723	724 [25]	ms, ri, std	18.6%
methylpyrazine (**9**)	817	819 [25]	ms, ri, std	<0.1%
2-methylbutyl acetate (**7**)	874	875 [25]	ms, ri, std	<0.1%
2,5-dimethylpyrazine (**10**)	903	908 [25]	ms, ri, std	1.5%
oct-1-en-3-ol (**3**)	974	974 [25]	ms, ri, std	<0.1%
octan-3-one (**8**)	982	979 [25]	ms, ri, std	<0.1%
trimethylpyrazine (**11**)	995	1000 [25]	ms, ri, std	0.2%
1,8-cineole (**13**)	1027	1026 [25]	ms, ri, std	8.5%
2-ethyl-3,6-dimethylpyrazine (**12**)	1074	1077 [24]	ms, ri, syn	<0.1%
veratrole (**20**)	1141	1141 [25]	ms, ri, std	0.2%
3,4-dimethylanisole (**23**)	1141		ms, std	0.2%
1,4-dimethoxybenzene (**22**)	1160	1161 [25]	ms, ri, std	0.2%
terpinen-4-ol (**18**)	1174	1174 [25]	ms, ri	0.1%
3-oxo-1,8-cineole (**17**)	1179	1186 [25]	ms, ri	0.7%
2β-hydroxy-1,8-cineole (**14**)	1208	1217 [26]	ms, ri	0.4%
2-oxo-1,8-cineole (**16**)	1213	1218 [27]	ms, ri	<0.1%
2α-hydroxy-1,8-cineole (**15**)	1220	1228 [26]	ms, ri	<0.1%
2,4,5-trimethylanisole (**24**)	1225		ms, syn	54.5%
1,2,3-trimethoxybenzene (**21**)	1308	1309 [28]	ms, ri, std	<0.1%
2,5-dimethyl-*p*-anisaldehyde (**25**)	1456		ms, std	0.5%
methyl 2,5-dimethyl-*p*-anisate (**26**)	1544		ms, syn	0.4%
1,8-dimethoxynaphthalene (**27**)	1657	1657 [19]	ms, ri	0.3%
pogostol (**19**)	1657	1651 [25]	ms, ri	0.3%

^a^Retention index *I* on a HP5-MS column. ^b^Identification based on ms: identical mass spectrum, ri: identical retention index, std: comparison to a commercially available standard compound, syn: comparison to a synthetic standard. ^c^Peak area in % of total peak area. The sum is less than 100%, because compounds originating from the medium, unidentified compounds and contaminants such as plasticisers are not mentioned.

Furthermore, a group of monoterpenes and the sesquiterpene alcohol pogostol (**19**) that was previously reported from other fungi [29–30] were observed. Monoterpenes were comprised of terpinen-4-ol (**18**), 1,8-cineole (**13**) as one of the major compounds in the extracts, and small amounts of its oxidation products 2β-hydroxy-1,8-cineole (**14**), 2α-hydroxy-1,8-cineole (**15**), 2-oxo-1,8-cineole (**16**) and 3-oxo-1,8-cineole (**17**). The monoterpene ether **13** has previously been reported from other *Hypoxylon* spp. [31] and the responsible monoterpene synthase has been identified [32]. Its hydroxylated derivatives **14** and **15** were found in insects feeding on leafs of *Melaleuca alternifolia* that contain large amounts of **13** [26], and both alcohols **14** and **15** along with the ketones **16** and **17** were reported as metabolites of **13** in human milk [27].

**Figure 4:**
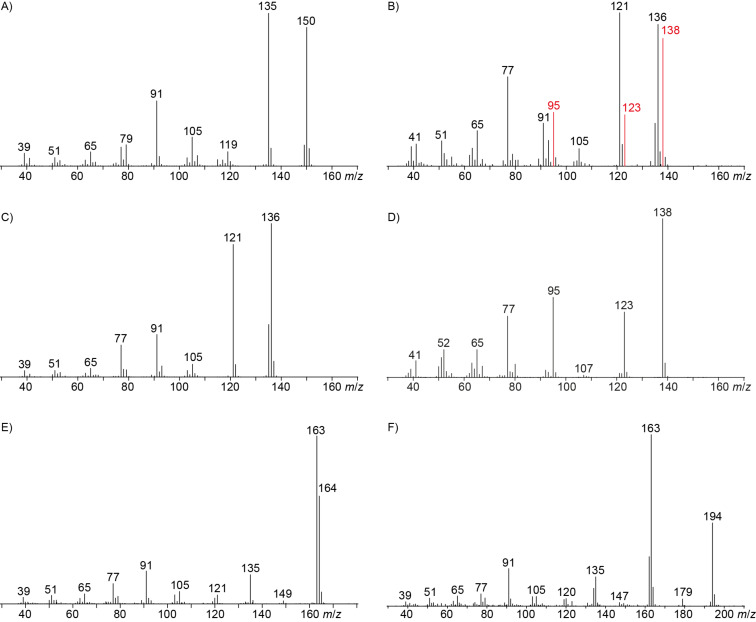
EI mass spectra of A) 2,4,5-trimethylanisole (**24**), B) the coeluting mixture of 3,4-dimethylanisole (**23**) and veratrole (**20**) with major peaks originating from **20** shown in red, C) the commercial standard of **23**, D) the commercial standard of **20**, E) 2,5-dimethyl-*p*-anisaldehyde (**25**), F) methyl 2,5-dimethyl-*p*-anisate (**26**).

**Scheme 1:**
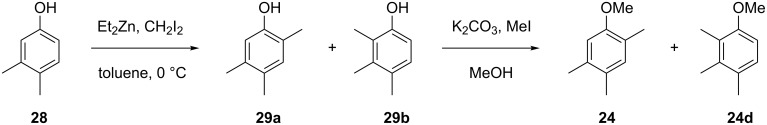
Synthesis of trimethylanisoles **24** and **24d**.

The mass spectrum of the main compound **24** from *H. griseobrunneum* (Figure 4A) showed several fragment ions in the low *m*/*z* region typical for an aromatic compound, while the fragment ion at *m*/*z* = 119 pointed to the loss of a methoxy group from the molecular ion ([M − 31]^+^), suggesting the structure of a trimethylanisole for **24**. Six constitutional isomers of this compound exist (Table 2). For four of these compounds the corresponding trimethylphenols were commercially available that were O-methylated with methyl iodide and K_2_CO_3_ to yield compounds **24a**, **24b**, **24c** and **24e**. The other two isomers 2,3,4-trimethylanisole (**24d**) and 2,4,5-trimethylanisole (**24**) were obtained by *ortho*-methylation of 3,4-dimethylphenol (**28**) via a known procedure [33], followed by HPLC purification of the products 2,4,5-trimethylphenol (**29a**) and 2,3,4-trimethylphenol (**29b**) and subsequent O-methylation (Scheme 1). Comparison of the GC retention index of the natural product (*I* = 1225) to the retention indices of all six standards narrowed the possible structures down to those of 2,4,5-trimethylanisole (*I* = 1225) and 2,3,5-trimethylanisole (*I* = 1227), while all other isomers could be ruled out. The final structural assignment of 2,4,5-trimethylanisole for **24** was based on the better matching mass spectrum of this compound in comparison to the alternative of **24c**. Compound **24** has not been reported from other natural sources before.

**Table 2:** Retention indices of all isomers of trimethylanisole.

structure	compound name^a^	*I*^b^

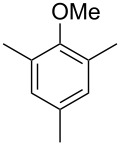	2,4,6-trimethylanisole (**24a**)	1157
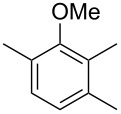	2,3,6-trimethylanisole (**24b**)	1181
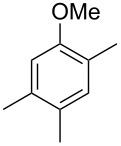	2,4,5-trimethylanisole (**24**)	1225
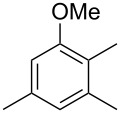	2,3,5-trimethylanisole (**24c**)	1227
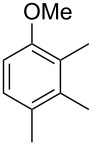	2,3,4-trimethylanisole (**24d**)	1257
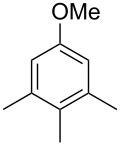	3,4,5-trimethylanisole (**24e**)	1271

^a^The natural product from *H. griseobrunneum* is **24**, its isomers are designated **24a**–**e**. ^b^Retention index *I* on a HP5-MS column.

**Scheme 2:**
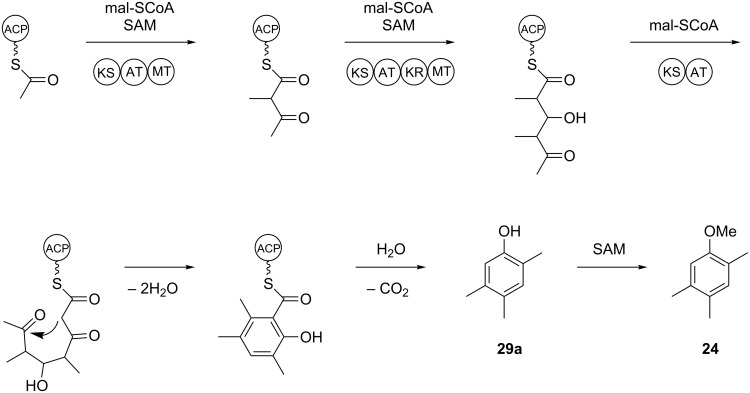
Hypothetical biosynthesis of **24**. ACP: acyl carrier protein, AT: acyl transferase, KR: ketoreductase, KS: ketosynthase, mal-SCoA: malonyl-SCoA, MT: methyl transferase, SAM: *S*-adenosyl-L-methionine.

**Figure 5:**
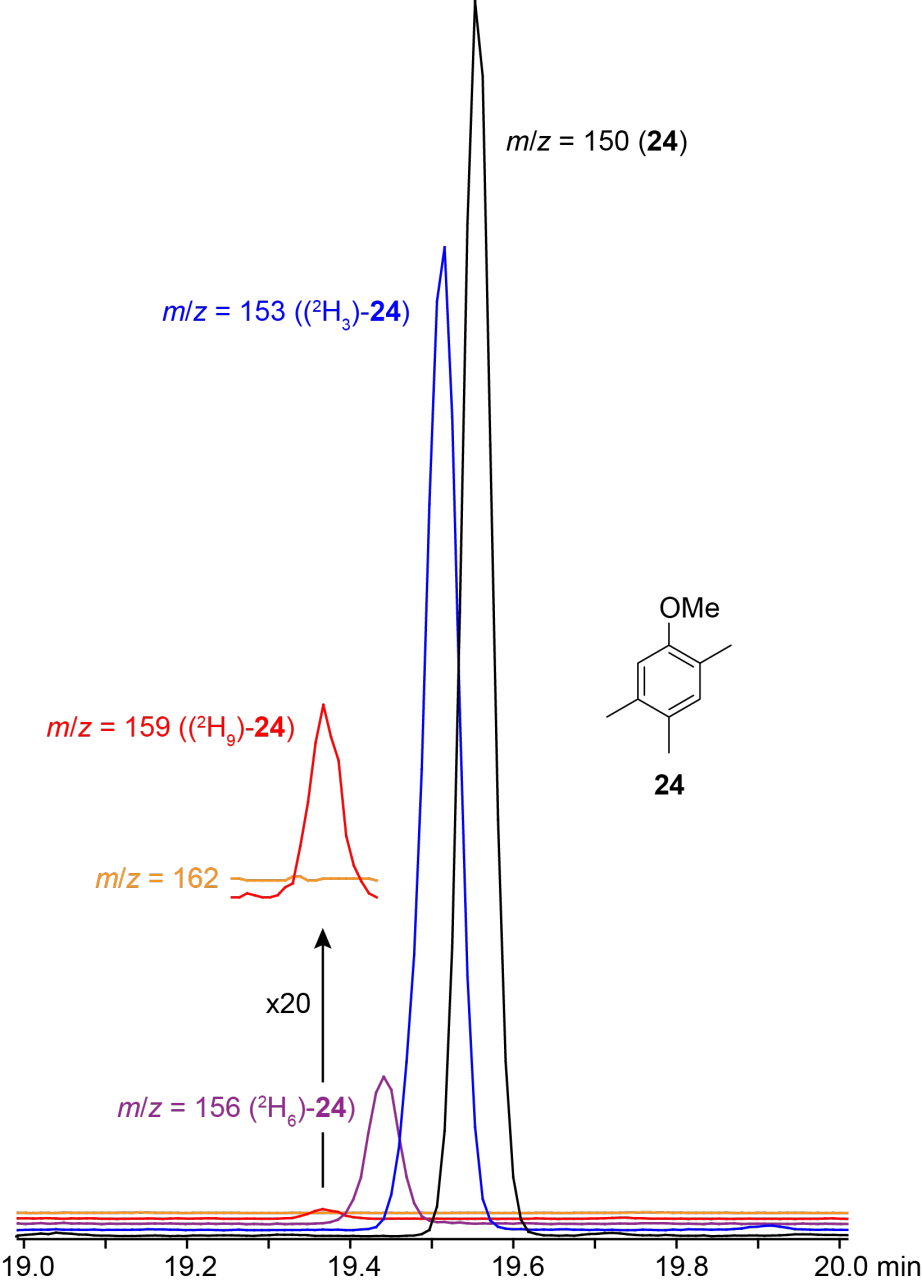
Biosynthesis of **24**. Feeding of (*methyl*-^2^H_3_)methionine resulted in the incorporation of labelling into up to three methyl groups of **24**. The shown ion trace chromatograms represent unlabelled **24** (black, *m*/*z* = 150), (^2^H_3_)-**24** (blue, *m*/*z* = 153), (^2^H_6_)-**24** (purple, *m*/*z* = 156), and (^2^H_9_)-**24** (red, *m*/*z* = 159). No incorporation into the fourth methyl group was observed (no peak visible for *m*/*z* = 162). For the ion trace chromatograms of *m*/*z* = 159 and 162 also expansions (20×) are shown.

The identification of **24** was further supported by a feeding experiment with (*methyl*-^2^H_3_)methionine. While the methylation pattern of the alternative structure **24c** is difficult to understand via a polyketide biosynthesis mechanism, the formation of the assigned structure of **24** by a polyketide synthase (PKS) can be easily rationalised (Scheme 2). The acetate starter unit, bound to the acyl carrier protein (ACP) of an iterative fungal PKS, can be elongated with malonyl-SCoA (mal-SCoA) followed by C-methylation with *S*-adenosyl-L-methionine (SAM). Two more rounds of elongation with mal-SCoA, the first extension with C-methylation and action of a ketoreductase (KR), result in a tetraketide intermediate that can be cyclised by aldol condensation, followed by elimination of water to result in the aromatic ring system. Thioester hydrolysis and decarboxylation produce **29a** that can be converted by SAM-dependent O-methylation into **24**. In summary, this hypothetical biosynthetic mechanism includes three SAM-dependent methylation steps. A feeding experiment with (*methyl*-^2^H_3_)methionine, the biosynthetic precursor of SAM, resulted in the incorporation of labelling into up to three methyl groups of **24**, but not into the fourth methyl group (Figure 5), which is in line with the biosynthetic model of Scheme 2. Note that because of an isotope effect the isotopomers of **24** can be separated by gas chromatography depending on their deuterium content [34–35], which makes the usage of (*methyl*-^2^H_3_)methionine superior to the usage of ^13^C-labelled methionine that would not have led to chromatographic separation of the isotopomers. In conjunction with the low incorporation rates obtained here, the results would have been difficult to interpret.

Another trace compound emitted by *H. griseobrunneum* showed a molecular ion at *m*/*z* = 136 and coeluted with exactly the same retention time as a second compound with a molecular ion at *m*/*z* = 138. In case of two coeluting compounds the individual compounds are often enriched in the right and left peak flanks, and their individual mass spectra can be extracted by careful background subtraction, but this was not the case here, so only the mass spectrum of the compound mixture was obtained (Figure 4B). The analysis of the observed fragment ions suggested that the compound with the molecular ion at *m*/*z* = 136 may be one of the isomers of dimethylanisole, explaining the fragment ion at *m*/*z* = 105 by the loss of the methoxy group ([M − 31]^+^), and in agreement with the 14 Da lower molecular ion in comparison to **24**. All six isomers of dimethylanisole were commercially available and a comparison of retention indices together with a personal inspection of the mixed mass spectrum of Figure 4B and the mass spectrum of 3,4-dimethylanisole (Figure 4C) unequivocally identified the natural product as 3,4-dimethylanisole (**23**, Table 3). Furthermore, the alternative structure of a trimethylphenol was ruled out, because all the isomers eluted later than **23** (Table 3). Interestingly, the elution order of the trimethylphenols is the same as for the corresponding trimethylanisoles with respect to their substitution patterns, and each trimethylphenol consistently elutes slightly later with an increase of the retention index by ca. 30–50 points than the trimethylanisole analogue (Table 2 and Table 3), which is explainable by the significantly higher polarity of the phenols compared to the anisoles. Compound **23** was recently reported from *Euphorbia golondrina* [36], but was never observed as a fungal natural product so far.

**Table 3:** Retention indices of all isomers of dimethylanisole and trimethylphenol.

structure	compound name^a^	*I*^b^

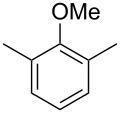	2,6-dimethylanisole (**23a**)	1056
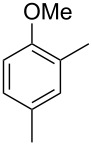	2,4-dimethylanisole (**23b**)	1103
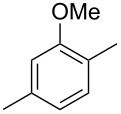	2,5-dimethylanisole (**23c**)	1104
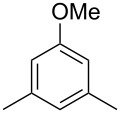	3,5-dimethylanisole (**23d**)	1114
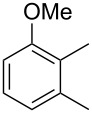	2,3-dimethylanisole (**23e**)	1128
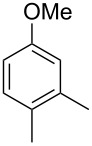	3,4-dimethylanisole (**23**)	1141
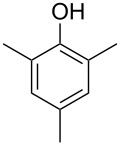	2,4,6-trimethylphenol (**23f**)	1198
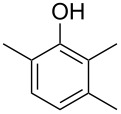	2,3,6-trimethylphenol (**23g**)	1227
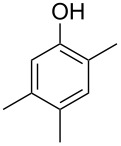	2,4,5-trimethylphenol (**23h**)	1262
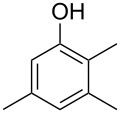	2,3,5-trimethylphenol (**23i**)	1267
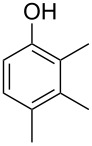	2,3,4-trimethylphenol (**23j**)	1296
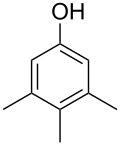	3,4,5-trimethylphenol (**23k**)	1311

^a^The natural product from *H. griseobrunneum* is **23**, its isomers are designated **23a**–**k**. ^b^Retention index *I* on a HP5-MS column.

Biosynthetically, the identified compound **23** can arise by a similar mechanism as discussed for **24**, potentially as a minor product of the same PKS, only the C-methylation step in the second round of chain extension needs to be skipped (Scheme 2). However, during the feeding experiment with (*methyl*-^2^H_3_)methionine the formation of **23** was suppressed, possibly because the additional supply of methionine resulted in a higher efficiency of the programmed methylation steps towards **24**.

The additional signals in the mixed mass spectrum (Figure 4B) at *m*/*z* = 138, 123 and 95 that do not originate from **23** are present with similar relative proportions as in the mass spectrum of veratrole (**20**, Figure 4D), and indeed a commercial standard of **20** revealed the same retention index of *I* = 1141 as the natural product, thus confirming the structure of veratrole for the second of the coeluting compounds. Its isomer 1,4-dimethoxybenzene (**22**) and a trimethoxybenzene **21** were also detected. Comparison to all three commercially available isomers of trimethoxybenzene established the identity of **21** as 1,2,3-trimethoxybenzene (Table 4). 1,8-Dimethoxynaphthalene (**27**) was also found and has been reported previously from other *Hypoxylon* spp. [19,37]. The corresponding compound 1,8-dihydroxynaphthalene is a known precursor of fungal melanin pigments [38].

**Table 4:** Retention indices of all isomers of trimethoxybenzene.

structure	compound name^a^	*I*^b^

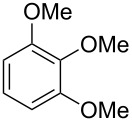	1,2,3-trimethoxybenzene (**21**)	1308
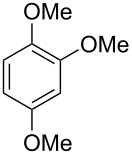	1,2,4-trimethoxybenzene (**21a**)	1368
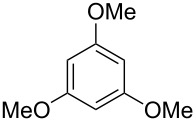	1,3,5-trimethoxybenzene (**21b**)	1409

^a^The natural product from *H. griseobrunneum* is **21**, its isomers are designated **21a** and **21b**. ^b^Retention index *I* on a HP5-MS column.

**Scheme 3:**
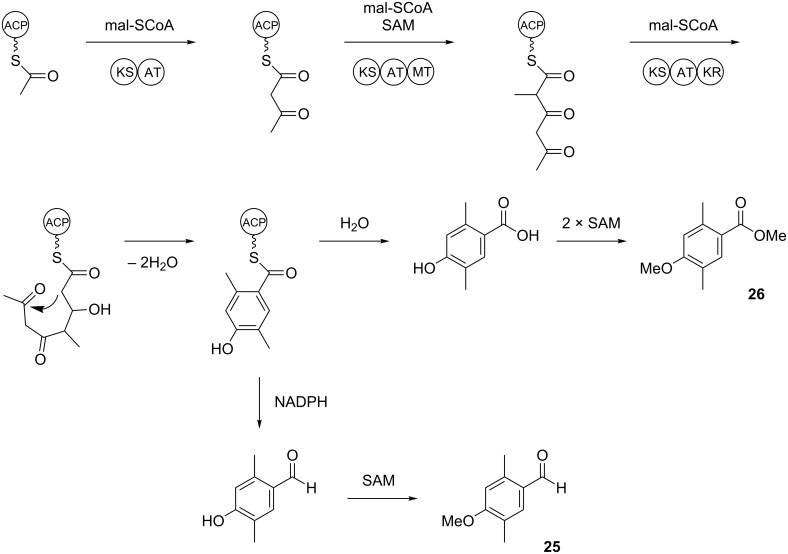
Hypothetical biosynthesis of **25** and **26**.

Two trace compounds exhibited the mass spectra shown in Figure 4E and Figure 4F that were similar to database spectra of 2,5-dimethyl-*p*-anisaldehyde (**25**) and methyl 2,5-dimethyl-*p*-anisate (**26**). The substitution pattern of these compounds is well explained by polyketide biosynthesis logic (Scheme 3). Starting from ACP-bound acetate, two non-reducing elongations with malonyl-SCoA, the first without and the second with C-methylation, followed by another elongation with reduction of the 3-oxo group and cyclisation yields the aromatic system of **25** and **26**. Hydrolytic cleavage from the ACP and two methylations of the phenol and the carboxylic acid result in **26**, while reductive cleavage and methylation of the phenol give **25**. The aldehyde **25** was commercially available and matched the natural product in terms of mass spectrum and retention time. Compound **25** was transformed into the corresponding methyl ester by treatment with iodine and potassium hydroxide in methanol [39]. The obtained material also showed identical behaviour in the GC–MS analysis to natural **26**. Both compounds **25** and **26** are new natural products.

### Identification of volatiles from *Hypoxylon macrocarpum*

**Figure 6:**
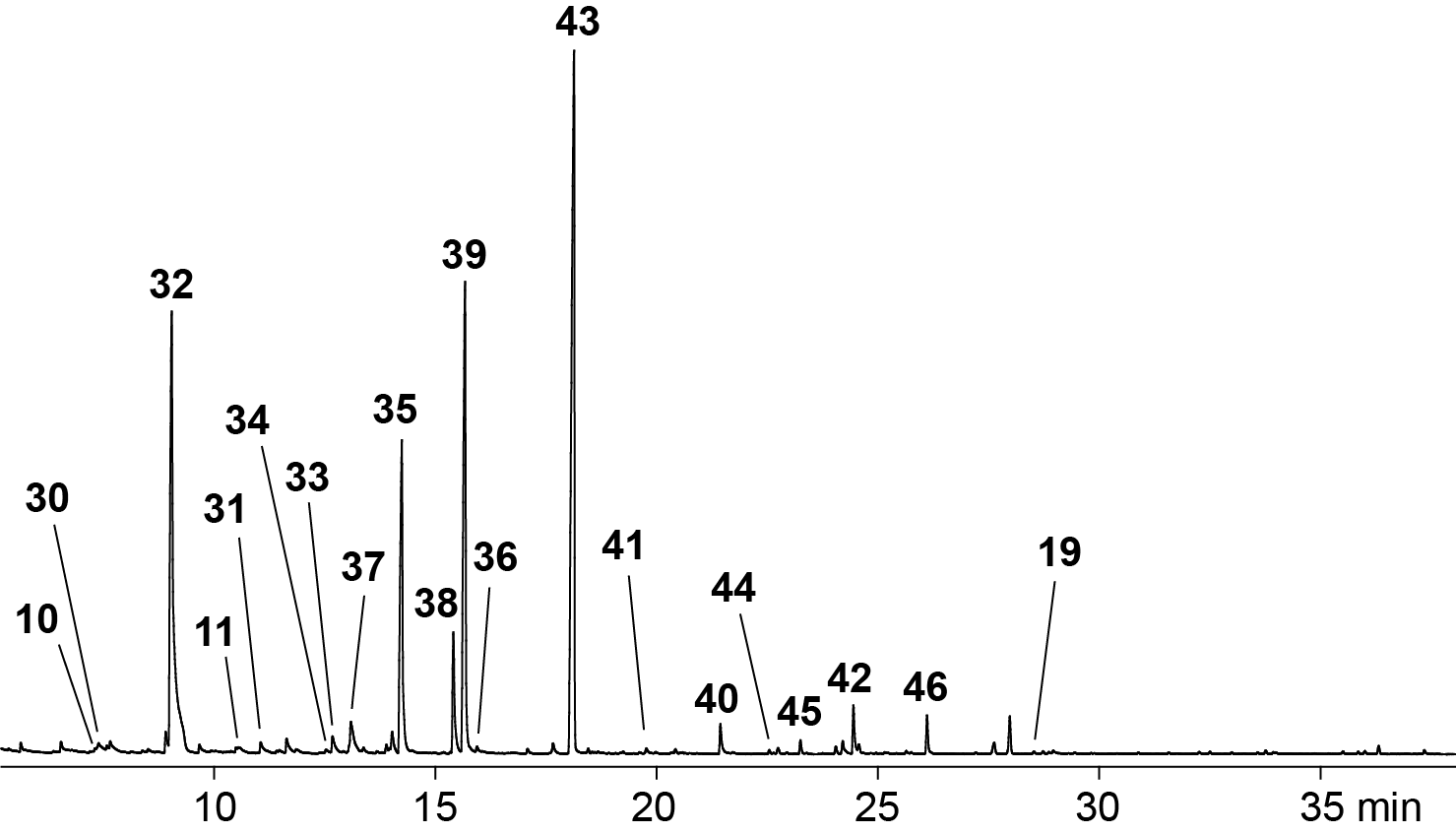
Total ion chromatogram of a CLSA headspace extract from *Hypoxylon macrocarpum* STMA 130423. Peak numbers refer to compound numbers in Table 5 and in Figure 7.

**Figure 7:**
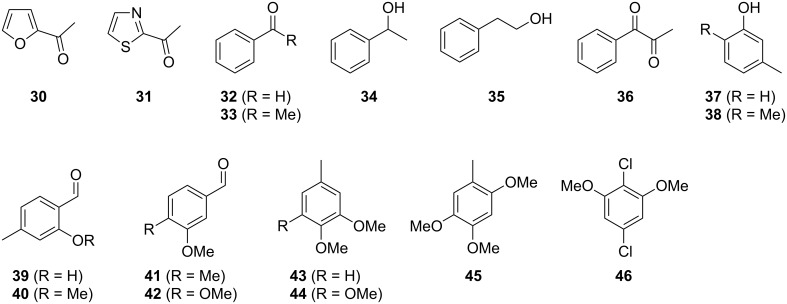
Volatiles from *Hypoxylon macrocarpum*.

The composition of the headspace extracts from *H. macrocarpum* (Figure 6 and Table 5) was completely different from the extracts of *H. griseobrunneum* with only the three compounds 2,5-dimethylpyrazine (**10**), trimethylpyrazine (**11**) and pogostol (**19**) being emitted by both species (Figure 7). The volatiles benzaldehyde (**32**) and 2-phenylethanol (**35**) as two of the main compounds, and the trace compounds 2-acetylfuran (**30**), 2-acetylthiazole (**31**), acetophenone (**33**), 1-phenylethanol (**34**), 1-phenylpropan-1,2-dione (**36**) and *m*-cresol (**37**) were readily identified from their mass spectra and retention indices and by comparison to authentic standards.

**Table 5:** Volatiles identified in headspace extract from *Hypoxylon macrocarpum* STMA 130423.

compound	*I*^a^	*I* (lit.)	identification^b^	peak area^c^

2,5-dimetylpyrazine (**10**)	903	908 [25]	ms, ri, std	0.1%
2-acetylfuran (**30**)	906	909 [25]	ms, ri, std	0.7%
benzaldehyde (**32**)	952	952 [25]	ms, ri, std	22.8%
trimethylpyrazine (**11**)	995	1000 [25]	ms, ri, std	0.5%
2-acetylthiazole (**31**)	1012	1014 [25]	ms, ri, std	0.6%
1-phenylethanol (**34**)	1054	1057 [25]	ms, ri, std	0.1%
acetophenone (**33**)	1059	1059 [25]	ms, ri, std	0.8%
*m*-cresol (**37**)	1071	1072 [25]	ms, ri	1.9%
2-phenylethanol (**35**)	1105	1106 [25]	ms, ri, std	11.6%
2,5-dimethylphenol (**38**)	1154	1152 [19]	ms, ri, std	3.6%
4-methylsalicylaldehyde (**39**)	1162	1165 [19]	ms, ri, std	16.4%
1-phenylpropan-1,2-dione (**36**)	1171	1175 [40]	ms, ri	0.1%
3,4-dimethoxytoluene (**43**)	1243	1240 [19]	ms, ri, std	29.1%
3-methoxy-4-methylbenzaldehyde (**41**)	1302	1307 [19]	ms, ri, std	0.2%
2-methoxy-4-methylbenzaldehyde (**40**)	1365	1364 [19]	ms, ri, std	0.9%
3,4,5-trimethoxytoluene (**44**)	1405		ms, std	0.1%
2,4,5-trimethoxytoluene (**45**)	1436		ms, syn	0.3%
3,4-dimethoxybenzaldehyde (**42**)	1483	1475 [25]	ms, ri, std	1.3%
2,5-dichloro-1,3-dimethoxybenzene (**46**)	1552	1556 [18]	ms, ri, std	1.0%
pogostol (**19**)	1657	1651 [25]	ms, ri	<0.1%

^a^Retention index *I* on a HP5-MS column. ^b^Identification based on ms: identical mass spectrum, ri: identical retention index, std: comparison to a commercially available standard compound, syn: comparison to a synthetic standard. ^c^Peak area in % of total peak area. The sum is less than 100%, because compounds originating from the medium, unidentified compounds and contaminants such as plasticisers are not mentioned.

**Figure 8:**
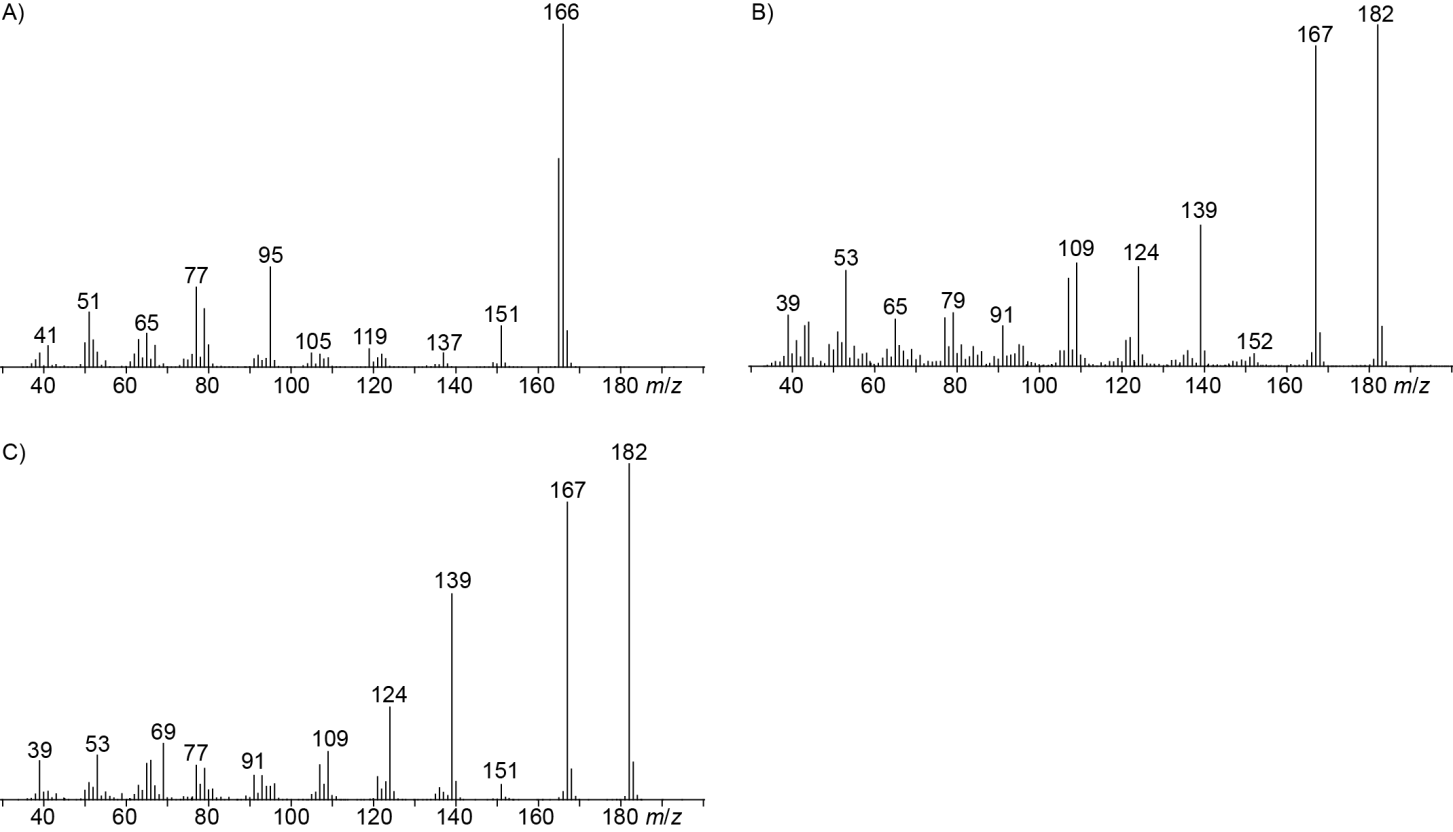
EI mass spectra of A) 3,4-dimethoxybenzaldehyde (**42**), B) 3,4,5-trimethoxytoluene (**44**), and C) 2,4,5-trimethoxytoluene (**45**).

The main compounds released by *H. macrocarpum* were identified as 3,4-dimethoxytoluene (**43**) and 4-methylsalicylaldehyde (**39**), while 2,5-dimethylphenol (**38**) and 2-methoxy-4-methylbenzaldehyde (**40**) were detected in lower amounts. All four compounds were previously observed in the bouquet of *H. invadens* and unambiguously identified by comparison to all possible isomers with different ring substitution patterns [19]. Furthermore, comparison to all ten isomers of methoxy-methylbenzaldehydes described in this study allowed for the identification of another trace compound from *H. macrocarpum* as 3-methoxy-4-methylbenzaldehyde (**41**). The chlorinated compound 2,5-dichloro-1,3-dimethoxybenzene (**46**) was also rigorously identified by comparison to all possible regioisomers that we had synthesised in a previous study [18]. Interestingly, the substitution pattern for the compound from *H. macrocarpum* is different to an isomer from the endophyte *Geniculosporium* sp. that was identified as 1,5-dichloro-2,3-dimethoxybenzene. Compound **46** has not been described as a natural product before. Another trace compound released by *H. macrocarpum* exhibited a mass spectrum that pointed to the structure of a dimethoxybenzaldehyde (Figure 8A). Comparison to all six commercially available isomers (Table 6) showed the identity of the natural product and 3,4-dimethoxybenzaldehyde (**42**).

**Table 6:** Retention indices of all isomers of dimethoxybenzaldehyde.

structure	compound name^a^	*I*^b^

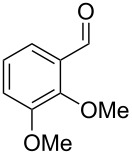	2,3-dimethoxybenzaldehyde (**42a**)	1391
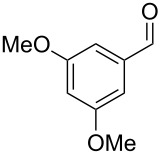	3,5-dimethoxybenzaldehyde (**42b**)	1445
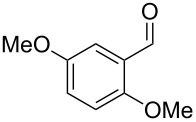	2,5-dimethoxybenzaldehyde (**42c**)	1468
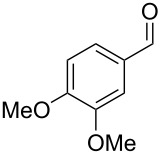	3,4-dimethoxybenzaldehyde (**42**)	1483
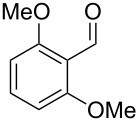	2,6-dimethoxybenzaldehyde (**42d**)	1531
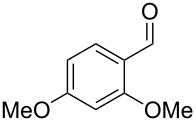	2,4-dimethoxybenzaldehyde (**42e**)	1543

^a^The natural product from *H. macrocarpum* is **42**, its isomers are designated **42a**–**e**. ^b^Retention index *I* on a HP5-MS column.

**Scheme 4:**
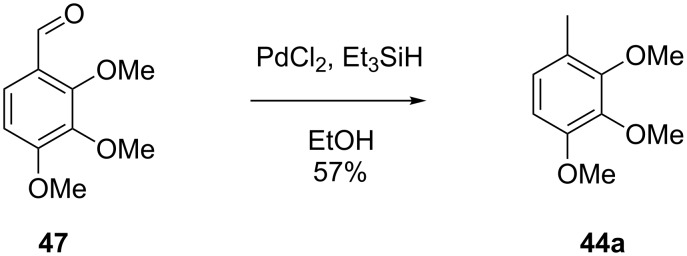
Synthesis of 2,3,4-trimethoxytoluene (**44a**).

Finally, two trace compounds with almost identical mass spectra (Figure 8B and Figure 8C), but clear separation by gas chromatography, were suggested to be trimethoxytoluenes. Two isomers, 3,4,5-trimethoxytoluene (**44**) and 2,4,6-trimethoxytoluene (**44d**), were commercially available. 2,3,4-Trimethoxybenzaldehyde (**47**) was reduced to 2,3,4-trimethoxytoluene (**44a**) using PdCl_2_ and Et_3_SiH [41] (Scheme 4), while the other three isomers were synthesised according to reported procedures [42–44]. Comparison of all six isomers to the two natural products (Table 7) resulted in their identification as 3,4,5-trimethoxytoluene (**44**) and 2,4,5-trimethoxytoluene (**45**). While **44** is a relatively widespread natural product, its isomer **45** has only once been tentatively identified by mass spectrometry in plants from the genus *Asarum* [45], but never from fungi before. However, it remains unclear how **45** was distinguished from **44** or other possible isomers in the earlier study.

**Table 7:** Retention indices of all isomers of trimethoxytoluene.

structure	compound name^a^	*I*^b^

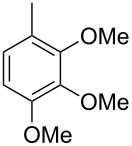	2,3,4-trimethoxytoluene (**44a**)	1321
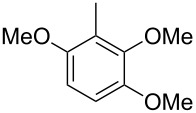	2,3,6-trimethoxytoluene (**44b**)	1397
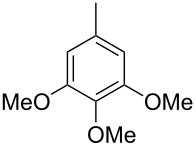	3,4,5-trimethoxytoluene (**44**)	1405
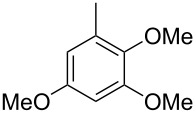	2,3,5-trimethoxytoluene (**44c**)	1410
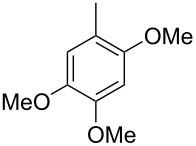	2,4,5-trimethoxytoluene (**45**)	1436
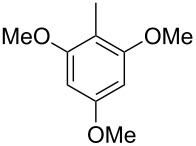	2,4,6-trimethoxytoluene (**44d**)	1488

^a^The natural products from *H. macrocarpum* are **44** and **45**, its isomers are designated **44a**–**d**. ^b^Retention index *I* on a HP5-MS column.

## Conclusion

Both investigated ascomycetes, *Hypoxylon griseobrunneum* and *Hypoxylon macrocarpum*, were found to emit complex mixtures of volatiles, mainly composed of aromatic compounds. As we have demonstrated, for unequivocal structural assignments based solely on GC–MS data it is important to compare the natural product to all possible constitutional isomers with different ring substitution patterns, because the mass spectra of these isomers are too similar to rely solely on MS data for compound identification. Therefore, also the retention index of the natural product must match the retention index of an authentic standard, and usually the retention indices of the isomeric aromatic compounds with different substitution patterns are sufficiently different for a confident structural assignment. Also biosynthetic considerations can help in the structure elucidation, because some aromatic substitution patterns are in line with a polyketide biosynthesis mechanism, while other substitution patterns may be difficult to understand. But such considerations should be made with care and should ideally be supported, e.g., by feeding experiments, as we have conducted in the present study. The main compounds of *H. griseobrunneum* were 2-methylbutan-1-ol, 1,8-cineol and 2,4,5-trimethylanisole, while *H. macrocarpum* released a completely different bouquet with the main compounds benzaldehyde, 2-phenylethanol, 4-methylsalicylaldehyde and 3,4-dimethoxytoluene. All these volatiles exhibit a characteristic smell and are likely main contributors to the odour produced by the fungi, but also some of the identified minor compounds may be important for the fungal fragrance. Notably, fungi of the genus *Hypoxylon* are interesting sources of new natural products, as exemplified by the identification of 2,4,5-trimethylanisole, 2,5-dimethyl-*p*-anisaldehyde and its corresponding methyl ester, and 2,5-dichloro-1,3-dimethoxybenzene. Therefore, it will be of high interest to investigate the volatiles from further *Hypoxylon* species in the near future.

## Experimental

### Strains and culture conditions

*Hypoxylon griseobrunneum* was obtained from a specimen collected in Martinique, Case Pilote, on a trail to Morne Venté on wood and bark of a dead dicotyledon branch in a mesophilic to xerophilic forest, on 25 August 2010 by Jacques Fournier [46]. A voucher specimen is deposited at the herbarium of the University of Lille, France (LIP, No MJF10120) and the culture is deposited with MUCL (Louvain-la Neuve, Belgium) under the accession number MUCL 53754.

*Hypoxylon macrocarpum* was obtained from ascospores of a specimen collected in Germany, Rhineland-Palatinate Province in the vicinity of Forst, near the Pechsteinkopf from wood of *Fagus* on 20 October 2012 by Benno and Marc Stadler [21]. A voucher specimen is deposited in the fungarium of the Helmholtz Centre for Infection Research (HZI, Braunschweig, Germany) under the accession number STMA 130423.

### Analysis of volatiles

The volatiles emitted by agar plate cultures of *H. griseobrunneum* and *H. macrocarpum* were collected through a closed-loop stripping apparatus (CLSA) [13] for ca. 1 day at room temperature and under natural light-dark rhythm. The CLSA charcoal filter traps were extracted with CH_2_Cl_2_ (50 μL, HPLC grade), followed by analysis of the extracts by GC–MS.

#### GC–MS

GC–MS analyses were performed with a 7890A GC coupled to a 5975C inert mass detector (Agilent, Hewlett-Packard Company, Wilmington, USA). The GC was equipped with a HP5-MS fused silica capillary column (30 m, 0.25 mm i. d., 0.25 μm film, Agilent). Conditions were inlet pressure: 77.1 kPa, He 23.3 mL min^−1^; injection volume: 1.5 μL; injector operation mode: splitless (60 s valve time); carrier gas: He at 1.2 mL min^−1^; GC program: 5 min at 50 °C, then increasing with 5 °C min^−1^ to 320 °C; transfer line 300 °C; electron energy 70 eV. Retention indices (*I*) were determined from a homologous series of *n*-alkanes (C_8_–C_38_).

### Synthesis of 2,4,5-trimethylphenol (**29a**) and 2,3,4-trimethylphenol (**29b**)

Diiodomethane (2.14 g, 8.0 mmol, 2 equiv) was dissolved in dry toluene (3 mL) under an argon atmosphere and the solution was cooled to 0 °C. To the vigorously stirred solution, Et_2_Zn in toluene (5.0 mL, 1.2 M, 6.0 mmol, 1.5 equiv) was added rapidly, followed immediately by the addition of 3,4-dimethylphenol (500 mg, 4.0 mmol) in toluene (3 mL). The reaction mixture was stirred at 0 °C for 5 min and then under reflux for 1.5 h. The reaction mixture was cooled to 0 °C and then quenched with an aqueous solution of NaHCO_3_ (10% w/w). The aqueous phase was extracted with diethyl ether for three times and the combined organic layers were dried over MgSO_4_. The solvent was removed under reduced pressure and the crude product was purified by column chromatography on silica gel (cyclohexane/ethyl acetate 5:1). The obtained product contained **29a** and **29b** as a mixture which was separated by HPLC (KNAUER Wissenschaftliche Geräte GmbH, Berlin, Azura; DAICEL Chiralpak IA column, 5 μm, 4.6 × 250 mm; hexane/2-propanol 95:5; retention times: 9.66 min (**29b**) and 10.89 min (**29a**)). The pure products were obtained as colourless liquids.

**2,4,5-Trimethylphenol (29a).** Yield: 14 mg (0.10 mmol, 3%). ^1^H NMR (500 MHz, CDCl_3_, 298 K) δ (ppm) 6.88 (s, 1H, CH), 6.59 (s, 1H, CH), 4.56 (br s, 1H, OH), 2.20 (s, 3H, CH_3_), 2.19 (s, 3H, CH_3_), 2.16 (s, 3H, CH_3_); ^13^C NMR (125 MHz, CDCl_3_, 298 K) δ (ppm) 151.6 (C_q_), 135.2 (C_q_), 132.1 (CH), 128.4 (C_q_), 120.5 (C_q_), 116.3 (CH), 19.4 (CH_3_), 18.7 (CH_3_), 15.2 (CH_3_).

**2,3,4-Trimethylphenol (29b).** Yield: 11 mg (0.08 mmol, 2%). ^1^H NMR (500 MHz, CDCl_3_, 298 K) δ (ppm) 6.87 (d, ^3^*J* = 8.1 Hz, 1H, CH), 6.56 (d, ^3^*J* = 8.1 Hz, 1H, CH), 4.54 (br s, 1H, OH), 2.22 (s, 3H, CH_3_), 2.20 (s, 3H, CH_3_), 2.19 (s, 3H, CH_3_); ^13^C NMR (125 MHz, CDCl_3_, 298 K) δ (ppm) 151.7 (C_q_), 136.6 (C_q_), 128.8 (C_q_), 127.5 (CH), 122.6 (C_q_), 112.0 (CH), 20.3 (CH_3_), 16.0 (CH_3_), 12.1 (CH_3_).

### Synthesis of trimethylanisoles **24** and **24a**–**e**

To a solution of the respective phenol derivative (**23f**–**k**, 15.0 mg, 0.11 mmol, 1 equiv) in dry DMF (2.2 mL), K_2_CO_3_ (15.2 mg, 0.11 mmol, 1 equiv) was added and the mixture was stirred at room temperature for 30 min. Methyl iodide (31 mg, 0.22 mmol, 2 equiv) was added and the reaction mixture was stirred at room temperature overnight. The reaction was quenched by addition of water and the aqueous phase was extracted three times with EtOAc. The combined organic layers were dried over MgSO_4_ and the solvent was removed under reduced pressure. The crude product was purified by column chromatography on silica gel (cyclohexane/ethyl acetate 20:1). The pure products were obtained as pale yellow liquids.

**2,4,5-Trimethylanisole (24)**. Yield: 5 mg (0.03 mmol, 32%). TLC (silica, cyclohexane/ethyl acetate 20:1): *R*_f_ = 0.48; ^1^H NMR (500 MHz, CDCl_3_, 298 K) δ (ppm) 6.89 (s, 1H, CH), 6.63 (s, 1H, CH), 3.80 (s, 3H, CH_3_), 2.23 (s, 3H, CH_3_), 2.17 (s, 3H, CH_3_), 2.16 (s, 3H, CH_3_); ^13^C NMR (125 MHz, CDCl_3_, 298 K) δ (ppm) 155.8 (C_q_), 134.6 (C_q_), 132.1 (CH), 128.0 (C_q_), 123.6 (C_q_), 112.1 (CH), 55.7 (CH_3_), 20.0 (CH_3_), 18.8 (CH_3_), 15.7 (CH_3_).

**2,4,6-Trimethylanisole (24a).** Yield: 6 mg (0.04 mmol; 36%). TLC (silica, cyclohexane/ethyl acetate 20:1): *R*_f_ = 0.31; ^1^H NMR (500 MHz, CDCl_3_, 298 K) δ (ppm) 6.82 (s, 2H, 2 × CH), 3.70 (s, 3H, CH_3_), 2.25 (s, 6H, 2 × CH_3_), 2.24 (s, 3H, CH_3_); ^13^C NMR (125 MHz, CDCl_3_, 298 K) δ (ppm) 154.9 (C_q_), 133.2 (C_q_), 130.6 (2 × C_q_), 129.5 (2 × CH), 59.9 (CH_3_), 20.8 (CH_3_), 16.1 (2 × CH_3_).

**2,3,6-Trimethylanisole (24b).** Yield: 7 mg (0.05 mmol; 42%). TLC (silica, cyclohexane/ethyl acetate 20:1): *R*_f_ = 0.42; ^1^H NMR (400 MHz, CDCl_3_, 298 K) δ (ppm) 6.92 (d, ^3^*J* = 7.6 Hz, 1H, CH), 6.83 (d, ^3^*J* = 7.6 Hz, 1H, CH), 3.70 (s, 3H, CH_3_), 2.27 (s, 3H, CH_3_), 2.24 (s, 3H, CH_3_), 2.20 (s, 3H, CH_3_); ^13^C NMR (100 MHz, CDCl_3_, 298 K) δ (ppm) 156.9 (C_q_), 136.0 (C_q_), 129.6 (C_q_), 128.2 (C_q_), 128.0 (CH), 125.3 (CH), 60.0 (CH_3_), 20.0 (CH_3_), 16.2 (CH_3_), 12.4 (CH_3_).

**2,3,5-Trimethylanisole (24c).** Yield: 8 mg (0.05 mmol; 48%). TLC (silica, cyclohexane/ethyl acetate 20:1): *R*_f_ = 0.47; ^1^H NMR (400 MHz, CDCl_3_, 298 K) δ (ppm) 6.62 (s, 1H, CH), 6.55 (s, 1H, CH), 3.81 (s, 3H, CH_3_), 2.30 (s, 3H, CH_3_), 2.24 (s, 3H, CH_3_), 2.11 (s, 3H, CH_3_); ^13^C NMR (100 MHz, CDCl_3_, 298 K) δ (ppm) 157.6 (C_q_), 137.7 (C_q_), 135.6 (C_q_), 123.1 (CH), 121.9 (C_q_), 109.0 (CH), 55.7 (CH_3_), 21.5 (CH_3_), 20.1 (CH_3_), 11.4 (CH_3_).

**2,3,4-Trimethylanisole (24d).** Yield: 5 mg (0.03 mmol, 32%). TLC (silica, cyclohexane/ethyl acetate 20:1): *R*_f_ = 0.54; ^1^H NMR (400 MHz, CDCl_3_, 298 K) δ (ppm) 6.96 (d, ^3^*J* = 8.3 Hz, 1H, CH), 6.64 (d, ^3^*J* = 8.3 Hz, 1H, CH), 3.79 (s, 3H, CH_3_), 2.23 (s, 3H, CH_3_), 2.18 (s, 3H, CH_3_), 2.17 (s, 3H, CH_3_); ^13^C NMR (100 MHz, CDCl_3_, 298 K) δ (ppm) 156.0 (C_q_), 136.4 (C_q_), 128.6 (C_q_), 127.2 (CH), 125.1 (C_q_), 107.8 (CH), 55.8 (CH_3_), 20.3 (CH_3_), 16.0 (CH_3_), 12.1 (CH_3_).

**3,4,5-Trimethylanisole (24e).** Yield: 8 mg (0.05 mmol; 48%). TLC (silica, cyclohexane: ethyl acetate = 20:1): *R*_f_ = 0.37; ^1^H NMR (400 MHz, CDCl_3_, 298 K) δ (ppm) 6.59 (s, 2H, 2 × CH), 3.77 (s, 3H, CH_3_), 2.27 (s, 6H, 2 × CH_3_), 2.11 (s, 3H, CH_3_); ^13^C NMR (100 MHz, CDCl_3_, 298 K) δ (ppm) 157.1 (C_q_), 137.7 (2 × C_q_), 127.2 (C_q_), 113.2 (CH), 55.3 (CH_3_), 21.0 (2 × CH_3_), 14.7 (CH_3_).

### Synthesis of methyl 2,5-dimethyl-*p*-anisate (**26**)

Similar to a reported procedure [39], 2,5-dimethyl-*p*-anisaldehyde (**25**, 1 g, 6.09 mmol, 1 equiv) was dissolved in MeOH (60 mL) and the solution was cooled to 0 °C. Solutions of KOH (1.045 g, 15.89 mmol, 2.6 equiv, in 20 mL MeOH) and I_2_ (2.01 g, 7.92 mmol, 1.3 equiv, in 10 mL MeOH) were added and the mixture was stirred for 90 min at 0 °C. The reaction was diluted with EtOAc, washed three times with saturated aqueous Na_2_S_2_O_3_ solution and subsequently with brine. The organic layer was dried over MgSO_4_ and the solvent was removed under reduced pressure. The crude product was purified via column chromatography (cyclohexane/ethyl acetate 10:1) on silica gel and the pure product was obtained as a colourless solid (277 mg, 1.43 mmol, 23%). TLC (silica, cyclohexane/ethyl acetate 3:1): *R*_f_ = 0.67. ^1^H NMR (500 MHz, CDCl_3_, 298 K) δ (ppm) 7.75 (s, 1H, CH), 6.64 (s, 1H, CH), 3.86 (s, 3H, CH_3_), 3.85 (s, 3H, CH_3_), 2.60 (s, 3H, CH_3_), 2.18 (s, 3H, CH_3_); ^13^C NMR (125 MHz, CDCl_3_, 298 K) δ (ppm) 167.9 (C_q_), 160.6 (C_q_), 140.8 (C_q_), 133.4 (CH), 123.9 (C_q_), 120.9 (C_q_), 112.9 (CH), 55.5 (CH_3_), 51.6 (CH_3_), 22.1 (CH_3_), 15.8 (CH_3_).

### Synthesis of 2,3,4-trimethoxytoluene (**44a**)

According to a known procedure [41], to a solution of 2,3,4-trimethoxybenzaldehyde (**47**, 500 mg, 2.55 mmol, 1 equiv) in EtOH (13 mL), SiEt_3_H (590 mg, 5.1 mmol, 2 equiv) was added under an argon atmosphere. PdCl_2_ (45.2 mg, 0.26 mmol, 10 mol %) was added and after stirring for 1 h, the reaction was quenched with H_2_O. The mixture was extracted three times with Et_2_O and the combined organic layers were dried over MgSO_4_. The solvent was removed under reduced pressure and the crude product was purified via column chromatography on silica gel (cyclohexane/ethyl acetate 10:1). The pure product was obtained as a colourless liquid (267 mg, 1.47 mmol, 57%). TLC (silica, cyclohexane/ethyl acetate 3:1): *R*_f_ = 0.50; ^1^H NMR (500 MHz, C_6_D_6_, 298 K) δ (ppm) 6.73 (dq, ^3^*J* = 8.4 Hz, ^4^*J* = 0.8 Hz, 1H, CH), 6.38 (d, ^3^*J* = 8.4 Hz, 1H, CH), 3.78 (s, 3H, CH_3_), 3.71 (s, 3H, CH_3_), 3.38 (s, 3H, CH_3_), 2.22 (d, ^4^*J* = 0.8 Hz, 3H, CH_3_); ^13^C NMR (125 MHz, C_6_D_6_, 298 K) δ (ppm) 152.9 (C_q_), 152.8 (C_q_), 143.5 (C_q_), 124.7 (CH), 124.3 (C_q_), 108.0 (CH), 60.6 (CH_3_), 60.3 (CH_3_), 55.8 (CH_3_), 15.9 (CH_3_).
